# Assessing Moderate to Vigorous Physical Activity in Older Adults: Validity of a Commercial Activity Tracker

**DOI:** 10.3389/fspor.2021.766317

**Published:** 2022-01-03

**Authors:** Brandon C. Briggs, Katherine S. Hall, Chani Jain, Madalina Macrea, Miriam C. Morey, Krisann K. Oursler

**Affiliations:** ^1^Geriatric Research and Education, Salem VA Medical Center, Salem, VA, United States; ^2^Department of Health and Human Performance, Concordia University Chicago, River Forest, IL, United States; ^3^Geriatric Research, Education, Clinical Center Durham Veterans Affairs Healthcare System, Durham, NC, United States; ^4^Center for the Study of Aging and Department of Medicine, Duke University Medical Center, Durham, NC, United States; ^5^Section of Pulmonary and Sleep, Salem VA Medical Center, Salem, VA, United States; ^6^Department of Medicine, University of Virginia School of Medicine, Charlottesville, VA, United States; ^7^Department of Medicine, Virginia Tech Carilion School of Medicine, Roanoke, VA, United States

**Keywords:** commercial activity device, validation study, daily step count, physical activity, older adults, ActiGraph accelerometer, Garmin Vivosmart, MVPA moderate to vigorous physical activity

## Abstract

**Purpose:** Despite the potential for commercial activity devices to promote moderate to vigorous physical activity (MVPA), limited information is available in older adults, a high-priority target population with unique gait dynamics and energy expenditure. The study purpose was to investigate the content validity of the Garmin Vivosmart HR device for step counts and MVPA in adults ≥65 years of age in free-living conditions.

**Methods:** Thirty-five participants (*M* age= 73.7 (6.3) years) wore Garmin and ActiGraph GT3X+ devices for a minimum of 2 days. Accuracy and intra-person reliability were tested against a hip worn ActiGraph device. Separate analyses were conducted using different accelerometer cut-off values to define MVPA, a population-based threshold (≥2,020 counts/minute) and a recommended threshold for older adults (≥1,013 counts/minute).

**Results:** Overall, the Garmin device overestimated MVPA compared with the hip-worn ActiGraph. However, the difference was small using the lower, age-specific, MVPA cut-off value [median (IQR) daily minutes; 50(85) vs. 32(49), *p* = 0.35] in contrast to the normative standard (50(85) vs. 7(24), *p* < 0.001). Regardless of the MVPA cut-off, intraclass correlation showed poor reliability [ICC (95% CI); 0.16(-0.40, 0.55) to 0.35(−0.32, 0.7)] which was supported by Bland-Altman plots. Garmin step count was both accurate (*M* step difference: 178.0, *p* = 0.22) and reliable [ICC (95% CI; 0.94) (0.88, 0.97)].

**Conclusion:** Results support the accuracy of a commercial activity device to measure MVPA in older adults but further research in diverse patient populations is needed to determine clinical utility and reliability over time.

## Introduction

Globally, the percentage of individuals aged 65 years and older is estimated to increase from 9.3% in 2020 to 16.0% in 2050 (United Nations Department of Economic Social Affairs, 2020). Despite the increase in longevity, there remains a gap in healthspan, translating to high comorbidity and disability in later years of life which could be improved by lifestyle factors (Queen et al., 2020). Physical activity is essential for healthy aging, preventing and improving chronic illness and enabling independent living. Moderate to vigorous physical activity (MVPA) is advocated for older adults (Chodzko-Zajko et al., 2009), although age-specific factors need to be considered (Zaleski et al., 2016). A systematic review found no evidence of increased risk for MVPA in the setting of cardiovascular disease (Kraus et al., 2019). However, adherence to the recommended 150 to 300 min of weekly MVPA is low among older adults (Watson et al., 2016). Recent activity guidelines emphasize that any MVPA can provide health benefits (Physical Activity Guidelines Advisory Committee, 2018), even MVPA accumulated in bouts of less than 10 min (Jakicic et al., 2019). Feasible strategies to increase physical activity, especially MVPA, are needed in older adults.

Wrist-worn consumer accelerometry devices are broadly available now, providing an opportunity to promote higher intensity physical activity in older adults. Validation work examining various devices across different populations is robust, yet there is a paucity of research in older adults. Several important knowledge gaps need to be addressed given the unique gait dynamics and energy expenditure of older adults (Schrack et al., 2016). In comparison with research-level accelerometers, consumer accelerometry devices are well-validated to measure step counts in both younger (Evenson et al., 2015) and older adults (Farina and Lowry, 2018). However, findings are less consistent in measurement of MVPA, and are particularly scarce in older adults (Straiton et al., 2018). There are established definitions of MVPA based on acceleration thresholds measured by research-grade accelerometers, such as ActiGraph devices, and a standard wear location of the hip. The threshold, or cut-off value in counts/minute, may need to be lower in older adults (Barnett et al., 2016) than the general population (Troiano et al., 2008) given differences in oxygen consumption at different walking speeds across age groups (Hall et al., 2013; Barnett et al., 2016). Yet, the bearing on commercial activity devices, and thus clinical utility in free-living conditions, is not clear. Validations studies of consumer devices in older adults should consider different thresholds to define MVPA to allow for the lower resting metabolic rate. In addition, consumer devices are routinely worn on the wrist, raising the question of the impact device wear location which could be amplified in older adults with an average slower gait.

We previously conducted a pilot study in adults ≥65 years of age to explore the acceptability and usability of consumer activity wrist devices from six manufactures and found Garmin Vivosmart HR to score the highest and most preferable (Tocci et al., 2016). The purpose of this cross-sectional study was to determine the content validity of the Garmin Vivosmart HR compared to ActiGraph GT3X+ for the domains of daily step count and MVPA in adults ≥65 years of age. We performed pair-wise comparisons of data collected during free-living conditions between the wrist-worn Garmin device and two ActiGraph devices simultaneously worn on both hip and wrist.

## Methods

### Participants

Male and female community-dwelling participants were recruited from an outpatient exercise program called Gerofit at the Salem VA Medical Center (VAMC). Gerofit is a national clinical program that includes individualized exercise prescriptions for Veterans ≥65 years of age and improves physical performance (Morey et al., 2018). Gerofit has minimal eligibility criteria; patients must be able to perform activities of daily living (ADL), follow instructions in a group setting, and be free of ischemic heart disease (angina) and severe lung disease (oxygen dependent). Gerofit patients who did not require a walking assist device and were able to provide written informed consent were eligible for participation in the study. Participants were recruited with posted flyers in the Gerofit exercise class and referred to research staff for more information. A convenience sample of 35 volunteers was selected.

### Procedures

The cross-sectional study was approved by the Salem VAMC Institutional Review Board (IRB) and Research and Development committees. All individuals provided written informed consent. Research staff established a Garmin Connect internet-based profile (https://connect.garmin.com/modern/) for each participant that included height, weight, sex, non-dominant hand, and year of birth, but no identifying information. Per Garmin instructions, gait length for each participant was estimated by measuring step count over 400 meters and then entered into the Garmin profile. Research staff placed a Garmin Vivosmart HR (Garmin International, Inc. Olathe, KS, USA) on the participant's non-dominant wrist after synching the device with the individual's Garmin Connect profile. The Garmin Vivosmart HR is a consumer-level activity tracker with a tri-axial accelerometer, barometric altimeter, and heart rate monitor. However, the heart rate option was turned off during the study to preserve battery life. Garmin data collection was continuous between device placement on day one and return on day four. Data from the Garmin device were uploaded to the Garmin Connect site by research staff, participants never had access. Results were generated by Garmin's proprietary software and included daily summary statistics on step count and minutes of physical activity by 3 intensities: light, active, or highly active. Cut-off values for intensities and wear time validation were not indicated or available from Garmin. Minutes in active and highly active periods were summarized and considered MVPA. Results from wear-day two and three were entered into an excel spread sheet for each participant.

ActiGraph (ActiGraph Pensacola, FL, USA) was selected as the research-grade device on the basis of strong validation evidence (Migueles et al., 2017). The GT3X+ is a tri-axial accelerometer that measures step count through the frequency and intensity of acceleration. Raw acceleration data was collected at 30 Hz with an epoch of 60 seconds. Two wear locations were chosen: the hip, a standard in adults, and the wrist, to directly compare with the Garmin wrist device. The ActiGraph hip device was attached via a waist belt clip to the non-dominant hip. The ActiGraph wrist device was attached via a Velcro wrist band to the non-dominant wrist below the Garmin device. Data from the two ActiGraph GT3X+ devices were uploaded and analyzed using the ActiLife 6.13 software (ActiGraph Pensacola, Florida, USA). ActiLife provides a location option for analysis and wrist was chosen where applicable. The Choi wear time validation algorithm was used and requires ≥10 hours per day of valid wear time (Choi et al., 2011). Moderate to vigorous physical activity (MVPA) in the vertical axis or vector magnitude (VM) was defined using three different cut-points: (1) ≥ 2,020 counts/minute per Troiano's definition for adults (Troiano et al., 2008); (2) ≥ 1,013 counts/minute per Barnett's definition for older adults (Barnett et al., 2016); (3) >1,924 VM counts/minute per Barnett's definition for older adults (Barnett et al., 2016). Data collected over two contiguous days (48 h) were analyzed to determine daily averages of step count and minutes of MVPA.

Participants were instructed to wear all activity devices continuously, including sleep, except for water activities such as bathing. Participants completed a wear-time log that noted any non-wear time or problems. Devices were placed and activated after the participant's last Gerofit exercise session of the week (Thursday or Friday) and collected from participants prior to their first exercise class of the following week (Monday or Tuesday). Each participant wore the activity trackers for a minimum of 48 consecutive hours while not participating in any structured Gerofit exercise.

### Statistical Analyses

The primary outcomes were daily average step count (steps/day) and duration of MVPA (minutes/day). For ActiGraph devices, the average was calculated as the mean of data collected over two calendar days from ActiLife software generated datafiles. Three measures of MVPA were calculated using the three different cut-off values in different respective ActiLife software analyses. For the Garmin device, the daily averages for each day were copied from the website.

Summary statistics and scatterplots were used to assess distribution of data and outliers. Lower than expected values were confirmed by review of the participant wear log and ActiLife wear time datafile. Data were tested for normal distribution by the Shapiro-Wilk test. The difference in outcomes between 2 devices was tested by signed rank test or two-sided paired *t*-test based on data distribution. Intraclass correlation coefficients (ICC) for continuous data were used as parameters for criterion validity using two-way mixed effects models and absolute agreement. ICC values within the 95% confidence interval that were less than 0.5, between 0.5 and 0.75, between 0.75 and 0.9, and greater than 0.90 were considered representative of poor, moderate, good, and excellent reliability, respectively (Koo and Li, 2016). Bland-Altman plots with limits of agreement (LoA) were used to provide a visual representation of the systematic differences between devices and to assess potential nonsystematic differences between devices. The Bland-Altman method calculates the mean difference between two methods of measurement (the ‘bias’) and 95% limits of agreement as the mean difference (2 standard deviations) (Myles and Cui, 2007). Thus, it is expected that 95% of differences between the two measurement methods will fall within the 95% limits of agreement.

## Results

Demographic characteristics and ambulatory function of the study population are summarized in Table 1. Although all participants completed the study and returned the devices, data was missing in some instances. GT3X+ hip data was incomplete (<10 h) in 4 participants; two forgot to reattach it after bathing the first morning, one had a broken waist clip, and one had low wear time. Among the remaining participants, the mean ±SD daily wear time was 17.6 ±4.9 h. GT3X+ wrist data was missing in one device. For the Garmin device, one participant had all data lost during download and another had missing intensity minutes.

**Table 1:** Description of the study population.

Characteristic^*^	**N (%) *n =* 35**
Age, years	73.7 (6.3)
Male	33 (94%)
Race	
African American	6 (17%)
Caucasian	28 (80%)
Other	1 (3%)
Body mass index (BMI), kg/m^2^	
Underweight and normal, <25	5 (14%)
Overweight, 25–29.9	14 (40%)
Obese, ≥30	16 (46%)
Gait speed, m/sec	1.25 (0.21)
6-MWD, meters	508.5 (117.7)

* *n (%) or mean (SD) for continuous measures. 6-MWD, six-minute walk distance*.

**Figure 1:**
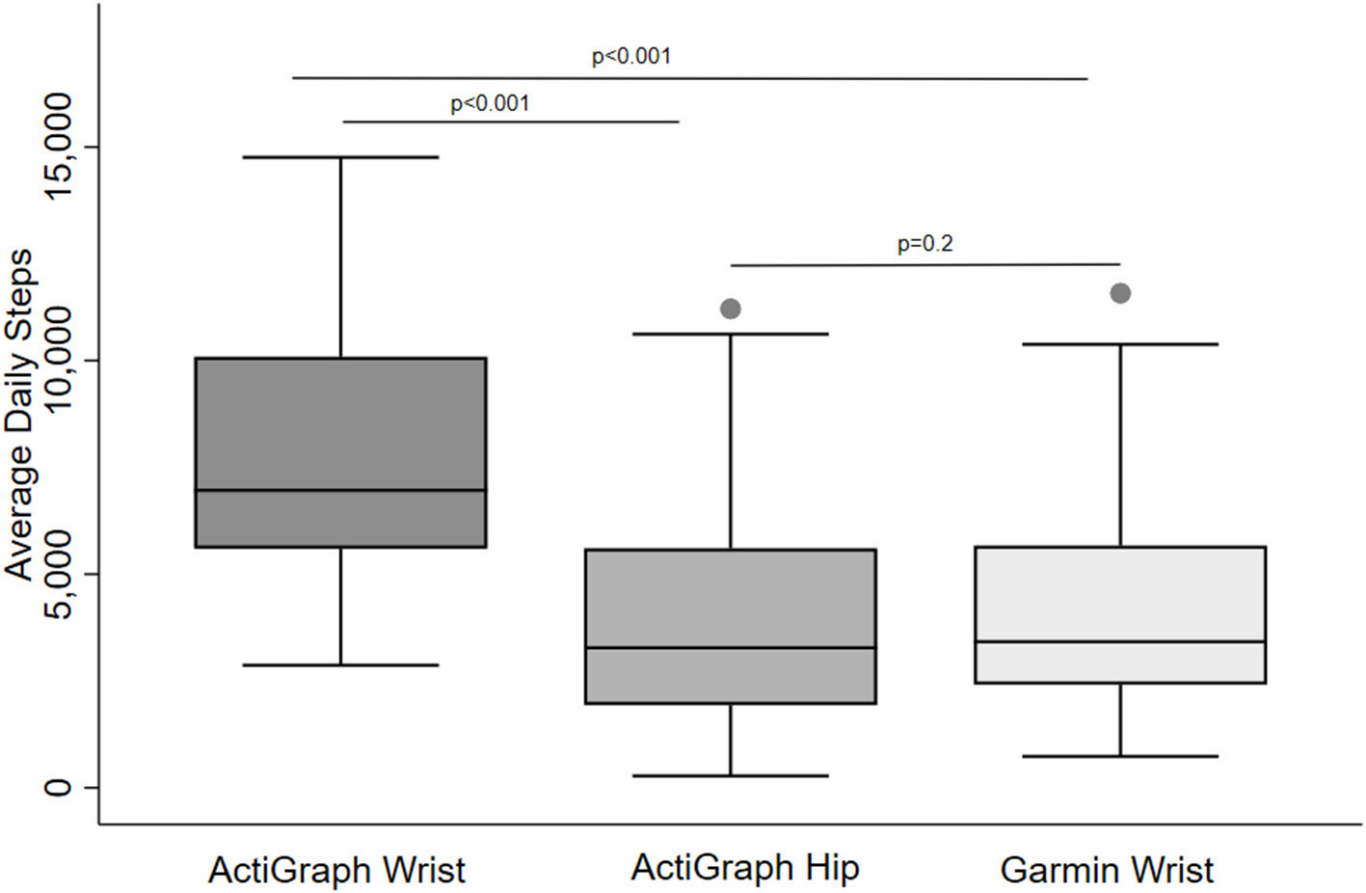
Daily step count in older adults collected by different activity devices and wear locations. Pair-wise comparisons were tested by the Signed Rank test.

Table 2 provides summary statistics of step count for each device as well as pair-wise test of differences. There was no significant difference in step count between the ActiGraph hip and Garmin devices. Daily step count collected by the ActiGraph wrist device was significantly higher than both ActiGraph hip device and the Garmin device (Figure 1). Similar results were found for the mean difference (bias) generated by Bland-Altman plots (Table 3). Correspondingly, the intraclass correlation (ICC) for daily step count showed good reliability between the Garmin and ActiGraph hip devices and moderate reliability in the other comparisons (Table 3).

**Table 2:** Daily steps and moderate vigorous physical activity difference between Garmin and ActiGraph devices.

	**Summary statistics**			**Pair-wise comparison between devices^†^**		
**Physical activity outcome**	**Garmin wrist**	**ActiGraph hip**	**ActiGraph wrist**	**Garmin wrist**	**Garmin wrist**	**ActiGraph wrist**
	***N =* 34**	***N =* 31**	***N =* 34**	**vs**.	**vs**.	**vs**.
				**ActiGraph**	**ActiGraph**	**ActiGraph**
				**hip**	**wrist**	**hip**
Step count, count/day						
Median (IQR)	3,419 (3254)	3,277 (3668)	6,963 (4491)	0.22	<0.001	<0.001
Min, Max	732, 11577	276, 11211	2,868, 14759			
MVPA, min/day (≥2,020 counts/min)^a^						
Median (IQR)	50 (85)^*^	7 (24)	52 (78)	<0.001	0.41	<0.001
Min, Max	0, 432	0, 86	9, 197			
MVPA, min/day (>1,013 counts/min)^b^						
Median (IQR)	50 (85)^*^	32 (49)	256 (137)	0.35	<0.001	<0.001
Min, Max	0, 432	1, 295	105, 533			
MVPA, min/day (>1,924 VM counts/min)^b^						
Median (IQR)	50 (85)^*^	38 (67)	275 (147)	0.79	<0.001	<0.001
Min, Max	0, 432	3, 303	128, 611			

*IQR, Interquartile Range (Quartile 3- Quartile 1); MVPA, moderate to vigorous physical activity; VM, vector magnitude*.

† *Signed Rank Test*.

* *MVPA data for Garmin device based on Garmin proprietary definitions*.

a *Troiano et al. (2008)*.

b *Barnett et al. (2016)*.

**Table 3:** Agreement between devices using Bland and Altman and intraclass correlation.

	**Paired device comparison**		
	**Garmin wrist**		**ActiGraph wrist**
**Physical activity**	**ActiGraph hip**	**ActiGraph wrist**	**ActiGraph Hip**
**Daily step count, count/day**			
Bland and Altman, Bias (LoA)	178.0 (−2431.6, 2787.5)	3620.0 (841.7, 6398.2)	−3845.2 (−6849.8, −840.6)
Intraclass correlation (95% CI)	0.94 (0.88, 0.97)	0.64 (−0.13, 0.90)	0.63 (−0.14, 0.89)
**MVPA, min/day (>2020 counts/min)** ^**a**^			
Bland and Altman, Bias (LoA)	57.5 (−113.8, 228.8)	−1.6 (−190.2, 187.0)	−59.5 (−164.0, 45.1)
Intraclass correlation (95% CI)	0.16 (−0.40, 0.55)	0.29 (−0.50, 0.66)	0.20 (−0.22, 0.54)
**MVPA, min/day (>1013 counts/min)** ^**b**^			
Bland and Altman, Bias (LoA)	−26.5 (−210.2, 157.2)	−210.1 (−460.7, 40.6)	−240.6 (−433.7, −47.4)
Intraclass correlation (95% CI)	0.35 (−0.32, 0.70)	0.10 (−0.14, 0.37)	0.10 (−0.10, 0.36)
**MVPA, min/day (>1924 VM counts/min)** ^**b**^			
Bland and Altman, Bias (LoA)	−19.2 (−206.4,167.9)	236.6 (−33.5, 506.6)	−259.8 (−454.6, −65.1)
Intraclass correlation (95% CI)	0.38 (−0.31, 0.71)	0.06 (−0.13, 0.30)	0.13 (−0.10, 0.44)

*ActiGraph GT3X+ model; Garmin Vivosmart HR model; LoA, limit of agreement; MVPA, moderate to vigorous physical activity, is based on defined cut-off values (Actigraph) or a commercial-derived value (Garmin)*.

a *Troiano et al. (2008)*.

b *Barnett et al. (2016)*.

**Figure 2:**
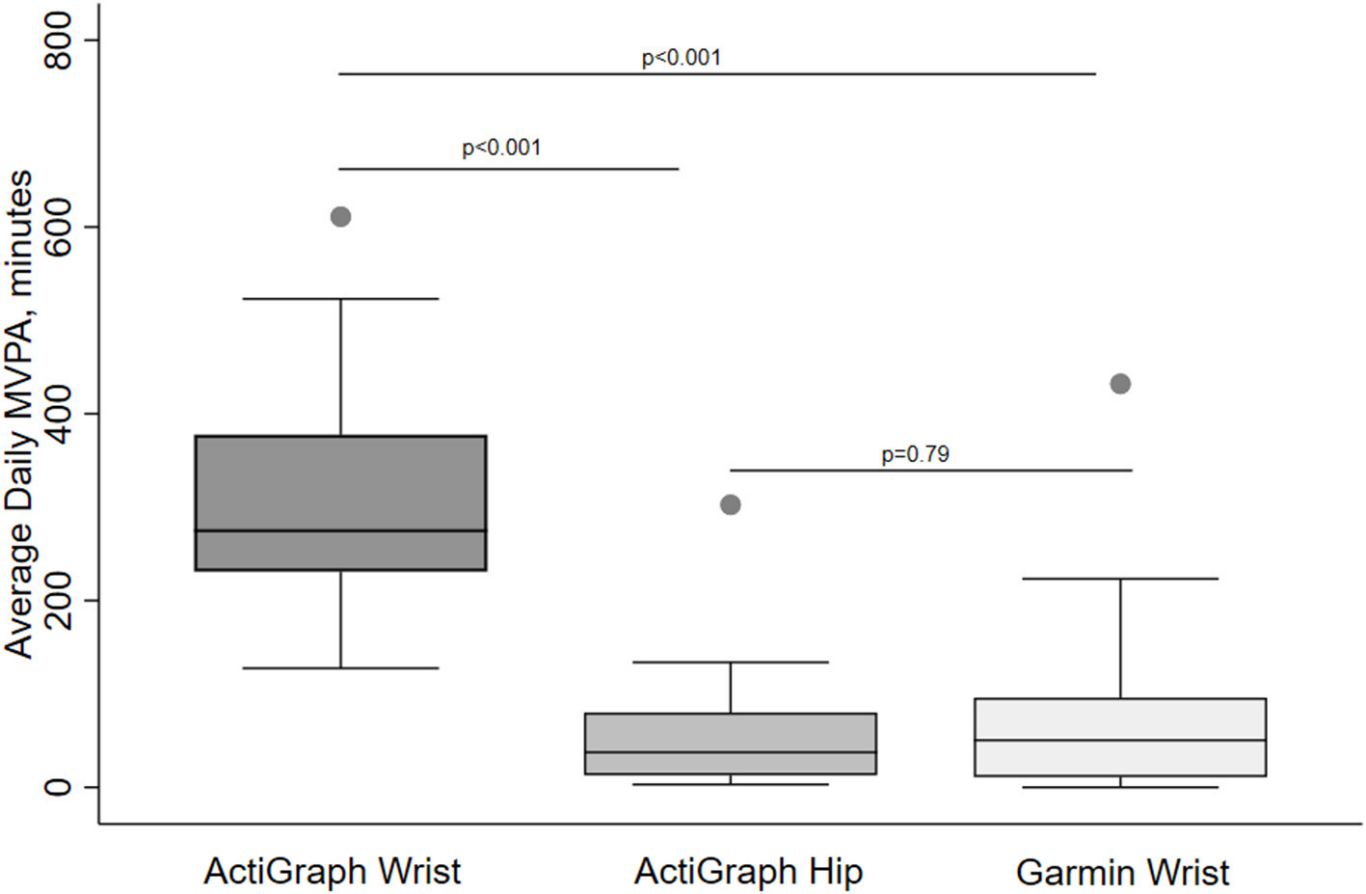
Daily minutes of moderate to vigorous physical activity (MVPA) in older adults collected by different activity devices and wear locations. MVPA is defined by >1,924 VM counts/min (ActiGraph) or Garmin proprietary definition. Pair-wise comparisons were tested by the Signed Rank test.

Table 2 provides MVPA results for each device using different accelerometer cut-off values to define MVPA. Overall, the Garmin device overestimated MVPA compared with the ActiGraph hip device, the reference standard. However, this difference was small and not statistically significant when a MVPA cut-off value for older adults was used. Specifically, Garmin MVPA was only 18 min higher than ActiGraph MVPA defined as ≥1,013 counts/min and only 12 min higher than ActiGraph MVPA defined as >1,924 vector magnitude counts/min. In contrast, daily Garmin MVPA was 43 min higher than the ActiGraph MVPA defined as of ≥ 2,020 counts/min (*p* < 0.001). Different results were found when the Garmin device was compared to the ActiGraph wrist-worn device. The Garmin underestimated daily MVPA by 4-fold compared to ActiGraph wrist MVPA data for older adults (*p* values < 0.01) but was identical to ActiGraph wrist MVPA data device defined as ≥ 2,020 counts/min. From another perspective, MVPA was consistently higher for the ActiGraph wrist-worn device compared to both Garmin and hip-worn ActiGraph devices, regardless of the MVPA cut-off value (Figure 2).

**Figure 3:**
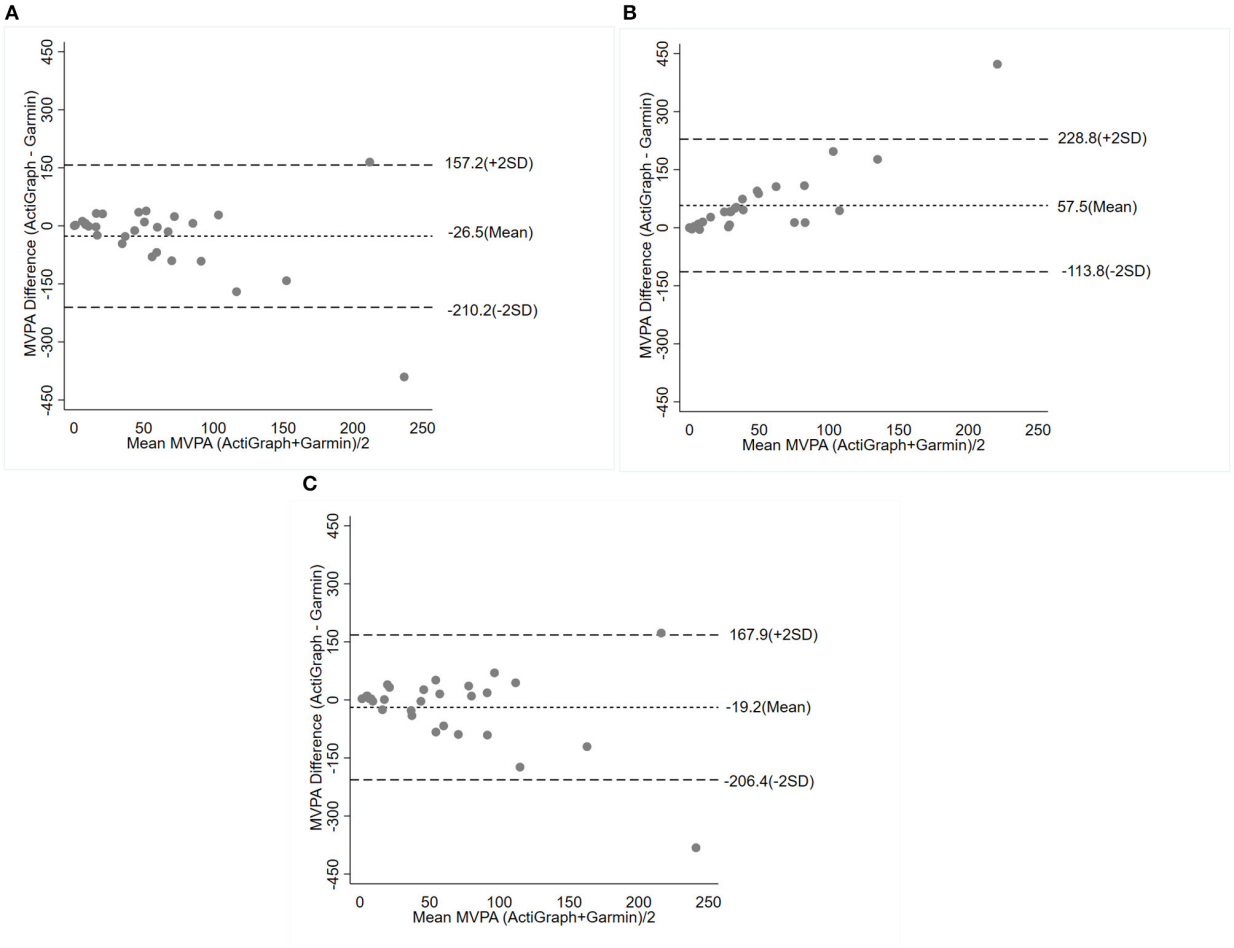
Bland-Altman plot of the mean difference (bias) and 95% limits of agreement for minutes per day of moderate to vigorous physical activity (MVPA) determined by ActiGraph and compared with Garmin Vivosmart. The results are presented based on the threshold for MVPA: ≥ 1,013 counts/minute **(A)**, 0≥ 2,020 counts/minute **(B)**, ≥ 1,914 VM counts/minute **(C)**. *Abbreviation*: VM, vector magnitude.

The mean difference in MVPA between devices (bias) derived from Bland-Altman plots supported these findings (Figure 3). The smallest difference in MVPA minutes between the Garmin device and the ActiGraph hip device was the comparison using vector magnitude counts for older adults. Yet, despite this relatively small difference, there was a wide level of agreement [mean bias (LoA) min; −19.3 (−206.5,167.9)]. Similarly, the reliability for MVPA minutes for this comparison was poor (ICC = 0.38), although it was the best amongst all device comparisons and cut-off values (Table 3).

## Discussion

The objective of this study was to investigate the accuracy and reliability of the Garmin Vivosmart HR, a consumer-level activity wrist device, to measure MVPA in older adults in a free-living condition. The results contribute to the limited knowledge of estimating MVPA in older adults with consumer activity devices. When compared to the ActiGraph hip device, a research standard, the Garmin overestimates MVPA. However, this difference was small if compared to MVPA defined by age-specific thresholds, supporting the accuracy of the Garmin device in older adults to measure higher intensity physical activity. Research in more diverse geriatric patient populations is needed given our select group of men without impaired ambulation. Further, the low intraclass correlation coefficient of MVPA data collected by this Garmin device incites overall caution for use of commercial devices in the research setting. In contrast, the Garmin device's measure of daily step count is both accurate and reliable. Future research and development, targeted to older adults, is warranted to facilitate usage of consumer activity devices to increase MVPA.

Most of the work in consumer activity devices focuses on young, healthy adults. To date, the capability of consumer devices to measure MVPA remains lower than step counts. But interpretation is limited by differences in proprietary definitions of MVPA for each company. Ferguson and colleagues compared several consumer-level devices worn on either hip or wrist with the ActiGraph GT3X+ on the hip in 21 healthy adults (mean age 33 years)(Ferguson et al., 2015). The reliability for MVPA (ICC range 0.36–0.79) was less than step counts (all ICC > 0.90), although a Garmin device was not included. A similar study in younger adults also found a wide range in MVPA reliability (ICC 0.37- 0.66) with all the consumer devices worn on the wrist (Degroote et al., 2018). Differences in minutes of daily MVPA, defined by the Freedson Adult cut-point ≥1952 counts/min, were small compared to the ActiGraph device. Among the different manufacturers, which did not include Garmin, the Fitbit MVPA data had the best accuracy and reliability.

The use of consumer activity devices in older adults is understudied, especially MVPA. In free-living conditions of older adults, devices from the manufacturer Fitbit (Fitbit Inc., San Francisco CA) are the most widely tested (Straiton et al., 2018). Studies with various Fitbit models (One, Zip, Flex, Charge) contain older adults with comorbid conditions (Paul et al., 2015; Farina and Lowry, 2018) including significant cardiac or pulmonary disease (Alharbi et al., 2016; Boeselt et al., 2016; Thorup et al., 2017). Yet, only one study reports MVPA (Alharbi et al., 2016). Alharbi and colleagues recruited physically active participants in either a cardiac rehabilitation program (*n* = 38) or a community walking program (*n* = 10) to evaluate the Fitbit Flex against the ActiGraph GT3X+ (Alharbi et al., 2016). While not targeting older adults, the mean (range) age was 65.6 (52–85) years. Similar to our findings, the commercial activity device overestimated the daily MVPA using the AcitGraph cut-off of 2,020 counts/min and Fitbit website-derived values. While information on reliability was not provided, the study classified individuals by their compliance with ≥150 min of MVPA per week and showed the Fitbit to be sensitive, though not specific, compared to the ActiGraph. This raises the question that physical activity measured by commercial activity devices could be misclassified as light rather than MVPA in older adults, which could lead to misguided physical activity counseling and intensity classification efforts. Previous research has shown substantial differences in the metabolic cost of daily activities by disability status among older adults, with lower-functioning individuals expending more energy to complete tasks than higher-functioning individuals (Knaggs et al., 2011). Thus, the potential for MVPA to be classified as light intensity is greater in an older population.

We chose the Garmin Vivosmart HR device for our validation study based on the preference of participants in Gerofit, who represent the end-user group of older adults most likely to be willing, and capable, of engaging in MVPA (Tocci et al., 2016). A recent study by Tedesco and colleagues in 20 older (>65 years) healthy adults also studied the performance of the Garmin Vivosmart against an ActiGraph device using the definition of ≥2,020 count/minute for MVPA (Tedesco et al., 2019). Our slightly larger study with longer wear time collaborates their finding of overestimated MVPA minutes for Garmin Vivosmart using this cut-off for MVPA. However, we also included age-specific thresholds for MVPA in our analyses and found no significant differences in this case. Our novel data in older adults suggest that MVPA estimated by commercial devices may be more accurate in older adults than younger adults if we assume that a lower threshold for MVPA better classifies higher intensity activity. This supposition is supported by laboratory-based research which shows resting metabolic rate (RMR) is reduced 32% in older adults and leads to misclassification of activity intensities for 60% of walking conditions (Hall et al., 2013). Further, oxygen consumption at different walking speeds in older adults wearing an ActiGraph device supports lower cut-off values for MVPA(Barnett et al., 2016). Lastly, our results for vector magnitude (VM) counts and comparison with the ActiGraph device worn on the wrist supports the capacity of the tri-axial accelerometer in the Garmin Vivosmart to differentiate arm and bodymovement.

Despite these encouraging results on accuracy, the ongoing challenge of the reliability of consumer activity devices to measure MVPA remains. While our finding on the accuracy of the Garmin Vivosmart for MVPA in older adults is consistent with some other brands in younger adults (Degroote et al., 2018), the intra-person reliability remains moderate at best. Our ICC results for the Garmin Vivosmart falls within the 95% confidence interval of the report by Tedesco et al. (2019) (ICC (95% CI); 0.68 (0.18, 0.91). Our comparison of Bland-Altman plots of MVPA shows a wide limit of agreement regardless of MVPA definition and demonstrates variability in device error. These results highlight the problem with relying on consumer activity devices to measure MVPA in the research setting. For the clinician, it is unknown if variability or inconsistency in MVPA over time would be noticeable to the patient, and if so, adversely affect the utility of the activity device as a motivational tool. However, in a survey of elderly adults (mean age 87 years), participants rate accuracy to be as important as usability (Hergenroeder et al., 2019). Ultimately, randomized trials are needed to determine the efficacy of commercial devices to increase MVPA in older adults.

With regards to daily step counts, our results show that the Garmin Vivosmart and ActiGraph hip device provide similar values with good reliability. Our findings are consistent with validation studies in older adults for other commercial activity devices (Straiton et al., 2018) in addition to a recent study with the Garmin Vivosmart (Tedesco et al., 2019). Much of the research to date in older adults includes relatively healthy older adults or those participating in an exercise program, such as our study. Further research is needed in older adults who have impaired gait. In a treadmill study of 10 commercial activity devices in young adults, slower walking speed decreased device validity (Fokkema et al., 2017). Of note, these results support the Garmin Vivosmart, which performed better at slow and average walking speeds compared to other commercial devices. However, our findings cannot be generalized to all older adults given our participants had an average gait speed of 1.25 m/sec. Gait dynamics in the setting of assistive walking devices, especially walkers, presents additional challenges and warrants specific consideration (Hergenroeder et al., 2019).

Further discussion of the generalizability of our results is warranted. The study population was predominantly men and may not represent women of the same age group. Further, all participants were patients engaged in a clinical center-based exercise program. Although devices were not worn on days when exercise classes were attended, the median duration of 50 min of daily MVPA is higher than most older adults. Results may be different in sedentary individuals and those using an assist device or with poorly controlled medical problems. Lastly, because the algorithm to classify intensity of physical activity in commercial devices is not available from manufactures, future derivations may be different and make it impossible to compare results.

We present new findings on the accuracy of a commercial activity device to measure MVPA in older adults when lower, age-specific cut-off values are used. Results support the use of a commercial activity device to estimate MVPA in older adults, but further research is needed to test if adherence with MVPA guidelines can be improved. Reliability of measuring MVPA remains problematic and hopefully will be addressed with advances in technology. The goal to use these popular activity devices to increase the intensity of physical activity in older adults will be advanced if manufacturers disclose their proprietary definitions for MVPA.

## Data Availability Statement

The datasets presented in this article are not readily available because datasets generated and analyzed during the current study are not publicly available due to information that could compromise research participant consent and anonymity. Limited data sets are available from the corresponding author on reasonable request and subject to permission from the Veterans Health Administration (VHA). Requests to access the datasets should be directed to krisann.oursler@va.gov.

## Ethics Statement

The studies involving human participants were reviewed and approved by Salem VAMC Institutional Review Board (IRB) and Research and Development Committees. The patients/participants provided their written informed consent to participate in this study.

## Author Contributions

BB, KO, and CJ analyzed the data and drafted the manuscript. KH, MM, and MCM provided critical revisions of this manuscript. All authors made substantial contributions in drafting or revising the manuscript and gave final approval of version submitted.

## Funding

Gerofit was supported by the Veterans Health Administration (VHA) Office of Geriatrics and Extended Care Non-institutional Long-Term Care and Mentored Partnership program and the VHA Office of Rural Health. This study was supported in part by VHA RRD I01 RX000667. MCM and KH supported in part by the Duke OAIC NIH/NIA AG028716. KH was supported in part by VHA RRD RX003120. Support for open access publication fees was provided by the Research and Development Service, Salem VA Medical Center. The funders had no role in any aspect of the research.

## Conflict of Interest

The authors declare that the research was conducted in the absence of any commercial or financial relationships that could be construed as a potential conflict of interest.

## Publisher's Note

All claims expressed in this article are solely those of the authors and do not necessarily represent those of their affiliated organizations, or those of the publisher, the editors and the reviewers. Any product that may be evaluated in this article, or claim that may be made by its manufacturer, is not guaranteed or endorsed by the publisher.
